# Supplementary material for: Genome-wide CRISPR screen for HSV-1 host factors reveals PAPSS1 contributes to heparan sulfate synthesis
Source: Commun Biol. 2022 Jul 19;5:694. doi: 10.1038/s42003-022-03581-9 (PMC9296583; doi:10.1038/s42003-022-03581-9)
Supplement: Supplementary file 2 — Supplementary Information [file 42003_2022_3581_MOESM2_ESM.pdf]

Genome-wide CRISPR screen for HSV-1 host factors reveals PAPSS1 contributes to heparan sulfate synthesis

Takeshi Suzuki[1], Yoshitaka Sato[1,2,*], Yusuke Okuno[3], Fumi Goshima[1], Tadahisa Mikami[4], Miki Umeda[1], Takayuki Murata[1,5], Yasuyuki Miyake[1], Takahiro Watanabe[1], Koichi Watashi[6,7,8,9], Takaji Wakita[6], Hiroshi Kitagawa[4] and Hiroshi Kimura[1,*]

[1]Department of Virology, Nagoya University Graduate School of Medicine, Nagoya 466-8550, Japan
[2]PRESTO, Japan Science and Technology Agency (JST), Kawaguchi 332-0012, Japan
[3]Department of Virology, Nagoya City University Graduate School of Medical Sciences, Nagoya 467-8601, Japan
[4]Laboratory of Biochemistry, Kobe Pharmaceutical University, Kobe 658-8558, Japan.
[5]Department of Virology and Parasitology, Fujita Health University School of Medicine, Toyoake 470-1192, Japan
[6]Department of Virology II, National Institute of Infectious Diseases, Tokyo 162-8640, Japan
[7]Research Center for Drug and Vaccine Development, National Institute of Infectious Diseases, Tokyo 162-8640, Japan
[8]Department of Applied Biological Sciences, Tokyo University of Science, Noda 278-8510, Japan
[9]Institute for Frontier Life and Medical Sciences, Kyoto University, Kyoto 606-8507, Japan

*Address correspondence to Yoshitaka Sato and Hiroshi Kimura, yssato@med.nagoya-u.ac.jp (YS); hkimura@med.nagoya-u.ac.jp (HK)

Supplementary Information

Supplementary Figure 1: Validation of the candidate genes.

Supplementary Figure 2: Perturbation of *MIR4647* does not suppress the cytopathic effect HSV-1 infection.

Supplementary Figure 3: *XYLT2* and *EXT2* are important for HepS biosynthesis in RPE-1 cells.

Supplementary Figure 4: *PAPSS1* contributes to the sulfation of HepS sugar chains in HAP1 cells.

Supplementary Figure 5: Confirmation of the absence of *PAPSS1*.

Supplementary Figure 6: Uncropped and unedited blot/gel images.

Supplementary Table 1: List of genes and miRNA enriched in our CRISPR screening.

Supplementary Table 2: HepS disaccharides analysis.

Supplementary Table 3: Primers used for RT-PCR.

Supplementary Table 4: List of sgRNA and their sequences.

Supplementary Table 5: Primers used for sgRNA cassette amplification and indexation.

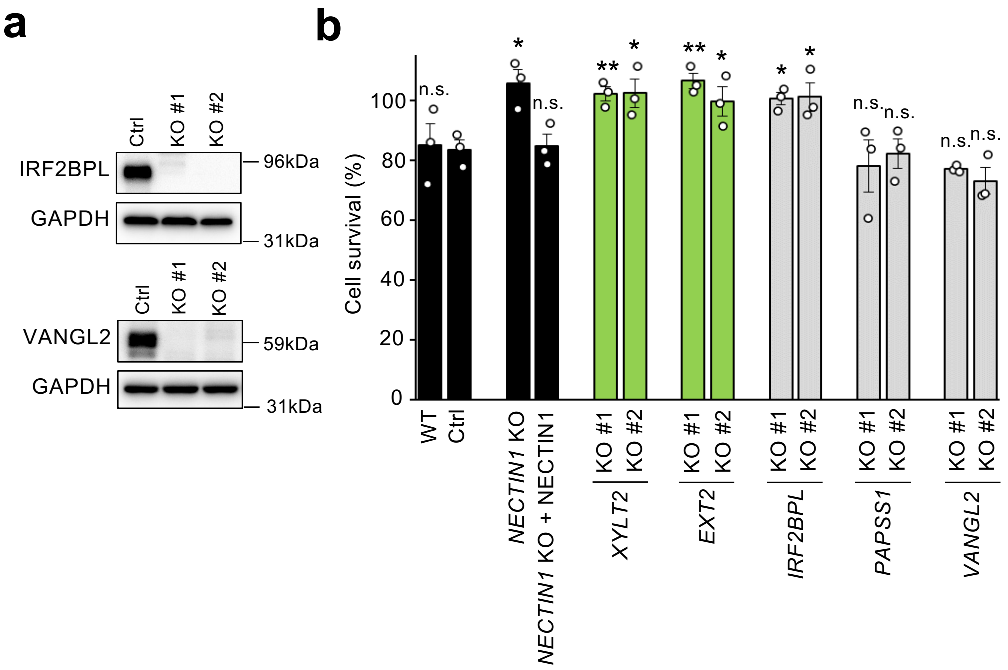

Supplementary Figure 1: Validation of the candidate genes.

(a) Western blotting confirming the absence of *IRF2BPL* or *VANGL2* expression in KO clones of HAP1 cells.

(b) WT, nontargeting Ctrl, and KO clones were infected with HSV-1 at a MOI of 0.1, after which their cell viabilities were measured at 72 hpi via the MTS assay. The results are presented as means ± SEM of three independent experiments.

Asterisks, $p < 0.05$; Double asterisks, $p < 0.01$; n.s., not significant.

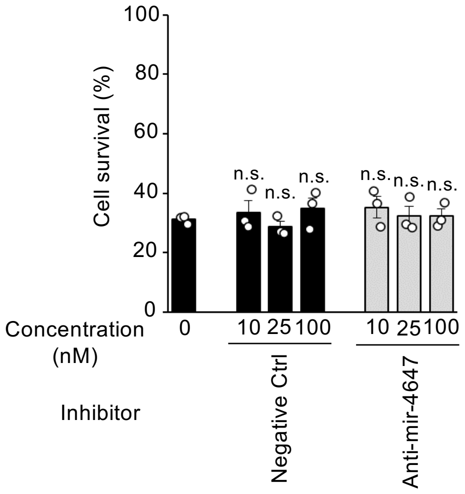

Supplementary Figure 2: Perturbation of *MIR4647* does not suppress the cytopathic effect HSV-1 infection. Wildtype HAP1 cells were treated with *miR-4647* or a negative control inhibitor at the indicated concentrations, followed by HSV-1 infection at a MOI of 3. The cell viabilities were measured at 48 hpi using the MTS assay. The results are presented as means ± SEM of three independent experiments. n.s., not significant.

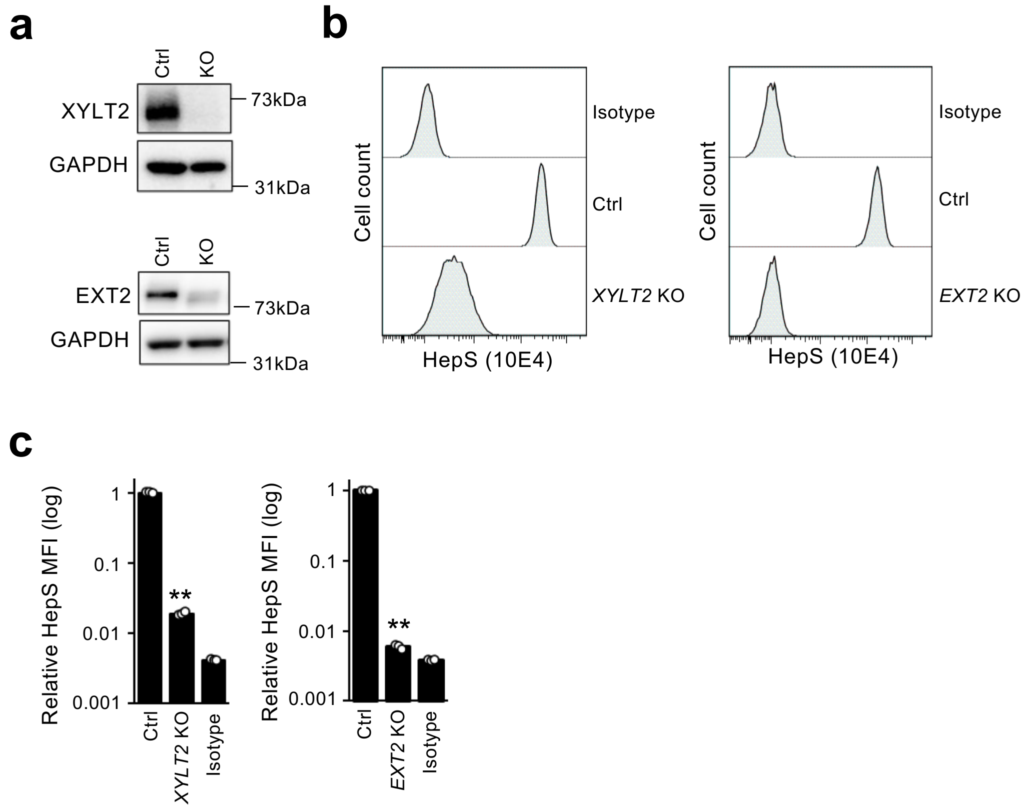

Supplementary Figure 3: *XYLT2* and *EXT2* are important for HepS biosynthesis in RPE-1 cells.
(a) Western blotting confirming the absence of *XYLT2* or *EXT2* expression in the KO clones of RPE-1 cells.
(b and c) HepS expression analysis by FACS (b). The graph summarizes the MFI of HepS expression analysis of three independent experiments (c). The results are presented as means ± SEM.
Double asterisks, $p < 0.01$.

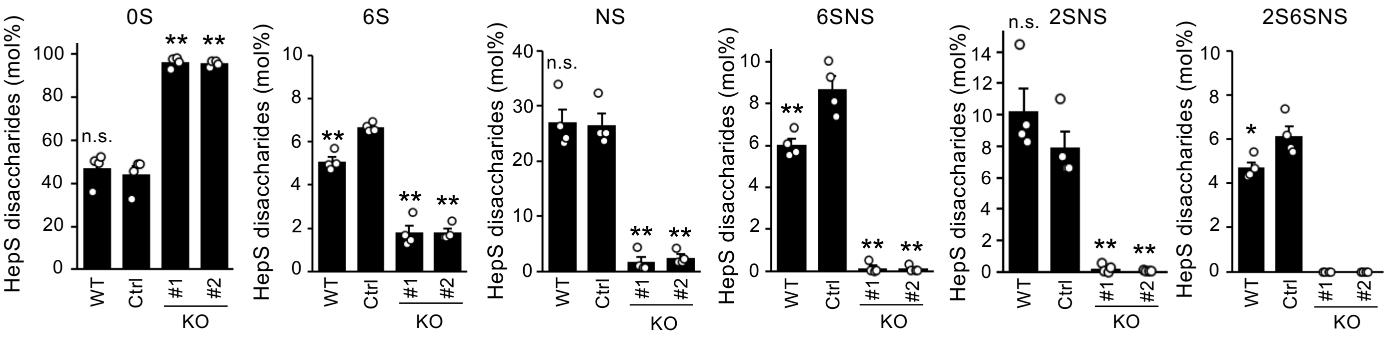

Supplementary Figure 4: *PAPSS1* contributes to the sulfation of HepS sugar chains in HAP1 cells. Disaccharide composition of wildtype, nontargeting control, and *PAPSS1*-KO clones of HAP1 cells. The results are presented as means ± SEM of four independent experiments. 0S, non-sulfated HepS disaccharide unit; 6S, 6-O-sulfated HepS disaccharide unit; NS, N-sulfated HepS disaccharide unit; 6SNS, 6-O-, N-disulfated HepS disaccharide unit; 2SNS, 2-O-, N-disulfated HepS disaccharide unit; 2S6SNS, 2-O-, 6-O-, N-trisulfated HepS disaccharide unit; Asterisks, $p < 0.05$; Double asterisks, $p<0.01$; n.s., not significant.

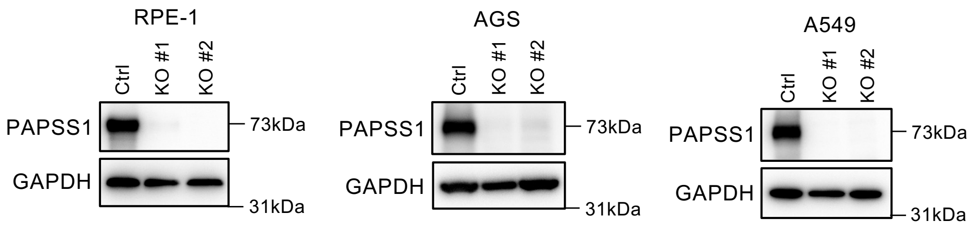

Supplementary Figure 5: Confirmation of the absence of *PAPSS1*.
Western blotting analysis confirming the absence of *PAPSS1* expression in the KO clones of human RPE-1, AGS, and A549 cells.

Supplementary Figure 6

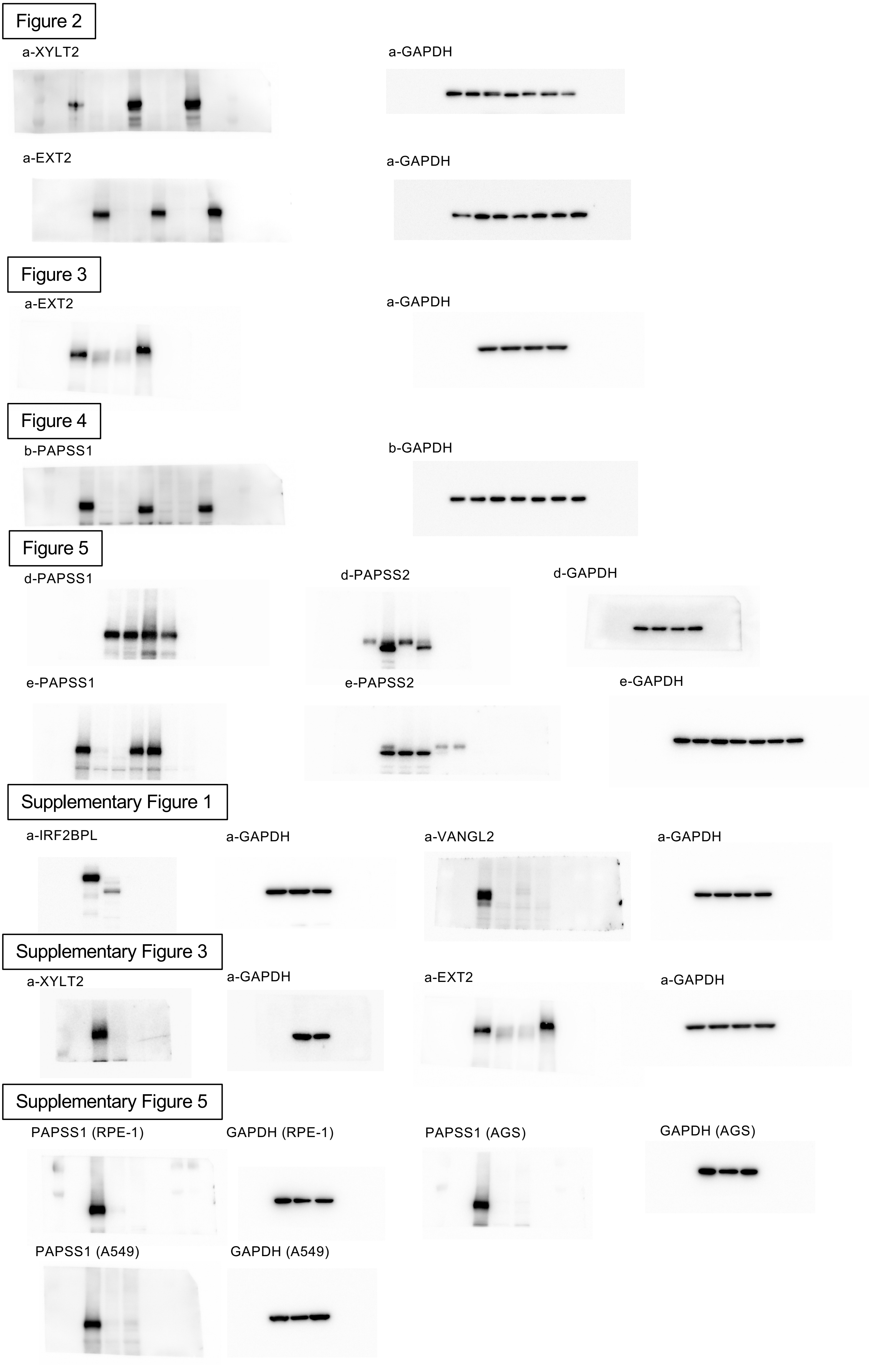

Supplementary Table 1: List of genes and miRNA enriched in our CRISPR screening.

Shaded columns of #: The already known genes in the heparan sulfate biosynthesis pathway. Genecards ID can be refferd to GeneCards: https://www.genecards.org/.

Gene Symbol	#	Genecards ID	Most enriched sgRNA (log10(RPM))	Second-most enriched sgRNA (log10(RPM))	Number of sgRNA (>150RPM)
NECTIN1		GC11M119624	5.094037	4.931866	5
B3GAT3		GC11M066689	5.032413	3.012237	4
EXT2		GC11P044095	4.763624	2.177548	2
XYLT2		GC17P050347	4.492601	3.991127	3
EXTL3		GC08P028602	4.356235	4.136489	4
hsa-mir-4647		GC06M045319	4.267369	3.656742	2
SLC35B2		GC06M044254	4.156771	3.920535	3
PAPSS1		GC04M107590	4.087792	4.033291	5
EXT1		GC08M117798	3.77241	3.457685	3
IRF2BPL		GC14M077024	2.723204	2.433825	2
B4GALT7		GC05P177600	2.539847	2.262761	2
VANGL2		GC01P160400	2.370367	2.200229	2

Supplementary Table 2: HepS disaccharides analysis

HAP1 (total HepS disaccharides)

WT (n=4) pmol per mg acetone powder

	1	2	3	4	Av	SD	mol%
0S	597.5	697.1	608.8	795.5	674.7	92.0	46.5
6S	81.8	65.6	70.5	75.9	73.5	7.0	5.1
NS	561.3	367.2	288.2	370.9	396.9	116.1	27.4
6SNS	100.2	77.4	84.7	87.2	87.4	9.5	6.0
2SNS	238.4	122.0	116.0	126.1	150.6	58.7	10.4
2S6SNS	71.7	61.6	66.8	71.2	67.8	4.7	4.7
Total	1651.0	1390.9	1235.0	1526.8	1450.9	178.9	100.0

Ctrl (n=4) pmol per mg acetone powder

	1.0	2.0	3.0	4.0	Av	SD	mol%
0S	498.3	543.7	542.7	595.2	545.0	39.6	43.1
6S	103.2	77.4	76.8	79.6	84.2	12.7	6.7
NS	497.7	299.7	263.2	306.8	341.8	105.6	27.0
6SNS	154.1	110.6	90.1	89.7	111.1	30.3	8.8
2SNS	169.0	88.0	73.0	80.8	102.7	44.6	8.1
2S6SNS	113.1	73.6	62.3	66.0	78.8	23.4	6.2
Total	1535.4	1193.0	1108.1	1218.1	1263.7	187.2	100.0

KO#1 (n=4) pmol per mg acetone powder

	1.0	2.0	3.0	4.0	Av	SD	mol%
0S	714.4	779.9	918.5	1007.8	855.1	132.6	96.2
6S	12.9	10.2	13.5	28.8	16.4	8.4	1.8
NS	34.3	7.5	6.1	7.3	13.8	13.7	1.6
6SNS	4.0	0.2	0.1	0.3	1.2	1.9	0.1
2SNS	4.3	0.6	0.2	3.2	2.1	2.0	0.2
2S6SNS	0.0	0.0	0.0	0.0	0.0	0.0	0.0
Total	770.0	798.4	938.4	1047.4	888.6	128.9	100.0

KO#2 (n=4) pmol per mg acetone powder

	1.0	2.0	3.0	4.0	Av	SD	mol%
0S	741.8	803.0	940.8	1005.8	872.9	121.6	95.6
6S	12.5	13.2	16.3	24.6	16.6	5.6	1.8
NS	33.6	13.9	17.4	24.2	22.3	8.6	2.4
6SNS	2.6	0.3	0.5	0.6	1.0	1.1	0.1
2SNS	1.0	0.5	0.5	0.5	0.6	0.3	0.1
2S6SNS	0.0	0.0	0.0	0.0	0.0	0.0	0.0
Total	791.5	830.8	975.5	1055.8	913.4	123.6	100.0

	WT	Ctrl	KO#1	KO#2
Av	1450.9	1263.7	888.6	913.4
SD	178.9	187.2	128.9	123.6

HAP1 (HepS disaccharide composition)

WT (n=4) disaccharide composition (mol%)

	1	2	3	4	Av	SD
0S	36.2	50.1	49.3	52.1	46.9	7.3
6S	5.0	4.7	5.7	5.0	5.1	0.4
NS	34.0	26.4	23.3	24.3	27.0	4.8
6SNS	6.1	5.6	6.9	5.7	6.1	0.6
2SNS	14.4	8.8	9.4	8.3	10.2	2.9
2S6SNS	4.3	4.4	5.4	4.7	4.7	0.5
Total	100.0	100.0	100.0	100.0	100.0	0.0

Ctrl (n=4) disaccharide composition (mol%)

	1	2	3	4	Av	SD
0S	32.5	45.6	49.0	48.9	44.0	7.8
6S	6.7	6.5	6.9	6.5	6.7	0.2
NS	32.4	25.1	23.7	25.2	26.6	3.9
6SNS	10.0	9.3	8.1	7.4	8.7	1.2
2SNS	11.0	7.4	6.6	6.6	7.9	2.1
2S6SNS	7.4	6.2	5.6	5.4	6.1	0.9
Total	100.0	100.0	100.0	100.0	100.0	0.0

KO#1 (n=4) disaccharide composition (mol%)

	1	2	3	4	Av	SD
0S	92.8	97.7	97.9	96.2	96.1	2.4
6S	1.7	1.3	1.4	2.7	1.8	0.7
NS	4.5	0.9	0.7	0.7	1.7	1.9
6SNS	0.5	0.0	0.0	0.0	0.1	0.3
2SNS	0.6	0.1	0.0	0.3	0.2	0.2
2S6SNS	0.0	0.0	0.0	0.0	0.0	0.0
Total	100.0	100.0	100.0	100.0	100.0	0.0

KO#2 (n=4) disaccharide composition (mol%)

	1	2	3	4	Av	SD
0S	93.7	96.7	96.4	95.3	95.5	1.3
6S	1.6	1.6	1.7	2.3	1.8	0.4
NS	4.2	1.7	1.8	2.3	2.5	1.2
6SNS	0.3	0.0	0.1	0.1	0.1	0.1
2SNS	0.1	0.1	0.0	0.0	0.1	0.0
2S6SNS	0.0	0.0	0.0	0.0	0.0	0.0
Total	100.0	100.0	100.0	100.0	100.0	0.0

HAP1 (HepS sulfation degree)

WT (n=4) sulfation degree

	1	2	3	4	Av	SD
0S	0.0	0.0	0.0	0.0	0.0	0.0
6S	5.0	4.7	5.7	5.0	5.1	0.4
NS	34.0	26.4	23.3	24.3	27.0	4.8
6SNS	12.1	11.1	13.7	11.4	12.1	1.2
2SNS	28.9	17.5	18.8	16.5	20.4	5.7
2S6SNS	13.0	13.3	16.2	14.0	14.1	1.5
Total	93.0	73.1	77.8	71.2	78.8	9.9

Ctrl (n=4) sulfation degree

	1	2	3	4	Av	SD
0S	0.0	0.0	0.0	0.0	0.0	0.0
6S	6.7	6.5	6.9	6.5	6.7	0.2
NS	32.4	25.1	23.7	25.2	26.6	3.9
6SNS	20.1	18.5	16.3	14.7	17.4	2.4
2SNS	22.0	14.8	13.2	13.3	15.8	4.2
2S6SNS	22.1	18.5	16.9	16.3	18.4	2.6
Total	103.3	83.4	77.0	76.0	84.9	12.7

KO#1 (n=4) sulfation degree

	1	2	3	4	Av	SD
0S	0.0	0.0	0.0	0.0	0.0	0.0
6S	1.7	1.3	1.4	2.7	1.8	0.7
NS	4.5	0.9	0.7	0.7	1.7	1.9
6SNS	1.0	0.1	0.0	0.1	0.3	0.5
2SNS	1.1	0.2	0.0	0.6	0.5	0.5
2S6SNS	0.0	0.0	0.0	0.0	0.0	0.0
Total	8.3	2.4	2.2	4.1	4.3	2.8

KO#2 (n=4) sulfation degree

	1	2	3	4	Av	SD
0S	0.0	0.0	0.0	0.0	0.0	0.0
6S	1.6	1.6	1.7	2.3	1.8	0.4
NS	4.2	1.7	1.8	2.3	2.5	1.2
6SNS	0.7	0.1	0.1	0.1	0.2	0.3
2SNS	0.3	0.1	0.1	0.1	0.1	0.1
2S6SNS	0.0	0.0	0.0	0.0	0.0	0.0
Total	6.7	3.4	3.7	4.8	4.7	1.5

	WT	Ctrl	KO#1	KO#2
Av	78.8	84.9	4.3	4.7
SD	9.9	12.7	2.8	1.5

* HepS sulfation degree = a + b + c + d + e + f

0S (mol%) x 0 = a
6S (mol%) x 1 = b
NS (mol%) x 1 = c
6SNS (mol%) x 2 = d
2SNS (mol%) x 2 = e
26NS (mol%) x 3 = f

Supplementary Table 3: Primers used for RT-PCR.

Target Gene		Sequence (5' to 3')
EXT2	F	ACAGGGATCTGATTCCTCC
	R	AATGACACGTTTCATAAGCTG
NECTIN1	F	ATCTTCCGGGCTGGTTTC
	R	ATACAGTAACACTAAAGCCAC
PAPSS1	F	TACCCTCCCGGCGCAGAGAAC
	R	ATGTGTCAAAGGTGGAGTGACTGG
XYLT2	F	GCAGGAAGATGGTGGCGAGC
	R	GGCCTCTGGTTCTTGCCTGTG

Supplementary Table 4: List of sgRNA and their sequences.

Target Gene	sgRNA No.	Sequence (5' to 3')
EXT2	1	TCTACGTGGACTTACCGGCA
	2	TCCGGTCATTGATAATCTGC
	3	GACCCGGGTAACCCACCAAC
IRF2BPL	1	ACTGTCTTGACCCCGACGGG
	2	GCAAGATTGTCTCCGGGACG
	3	CGCCGCTTCCTTAGCCGACA
NECTIN1	1	TTCTCGATTGCCCGTAGGGA
	2	GAATTCCACACGCTCGCGGT
	3	AATTCCACACGCTCGCGGTA
PAPSS1	1	TGAGTCGCAGTCAGAACTAT
	2	GATGTATGAGGGCCGCCGTG
	3	TGCGAAGAATGGCCACACGG
PAPSS2	1	ACTTGACCACGTCCGAGCTG
	2	TTTCAGCCTCAGCTCGGACG
	3	TTTCCGGCACAAAGAGTTCG
VANGL2	1	GCAGTTCGCCGTGTCGCTGG
	2	GGTGCAGTTCGCCGTGTCGC
	3	GCCAACGTTGTAGAAGCGGC
XYLT2	1	TGTAGGCGATTCGCACCGGG
	2	ATGTAGGCGATTCGCACCGG
	3	CATGTAGGCGATTCGCACCG
Nontargeting control		GCACTACCAGAGCTAACTCA

Supplementary Table 5: Primers used for sgRNA cassette amplification and indexation.

Round	Primer name	Sequence (5' to 3')
1st	Gecko2ins-F-15OL	AGACGTGTGCTCTTCCGATCTTCTTGTGGAAAGGACGAAACACCG
1st	Gecko2ins-R-15OL	TACACGACGCTCTTCCGATCTTGTGGGCGATGTGCGCTCTG
2nd	P5-15OL	AATGATACGGCGACCACCGAGATCTACACTCTTTCCCTACACGACGCTCTTC
2nd	P7-15OL-Index17	CAAGCAGAAGACGGCATACGAGATCTCTACGTGACTGGAGTTCAGACGTGTGCTCTTC
2nd	P7-15OL-Index18	CAAGCAGAAGACGGCATACGAGATGCGGACGTGACTGGAGTTCAGACGTGTGCTCTTC
2nd	P7-15OL-Index19	CAAGCAGAAGACGGCATACGAGATTTTCACGTGACTGGAGTTCAGACGTGTGCTCTTC
2nd	P7-15OL-Index20	CAAGCAGAAGACGGCATACGAGATGGCCACGTGACTGGAGTTCAGACGTGTGCTCTTC
