# Supplementary material for: Genome-wide CRISPR screen for HSV-1 host factors reveals PAPSS1 contributes to heparan sulfate synthesis
Source: Commun Biol. 2022 Jul 19;5:694. doi: 10.1038/s42003-022-03581-9 (PMC9296583; doi:10.1038/s42003-022-03581-9)
Supplement: Supplementary file 3 — Description of Additional Supplementary Files [file 42003_2022_3581_MOESM3_ESM.pdf]

Description of Additional Supplementary Files

File name: Supplementary Data 1
Description: Read counts of next generation sequencing

File name: Supplementary Data 2
Description: The source data behind the graphs in the paper
