# Supplementary material for: Genome-wide CRISPR screen for HSV-1 host factors reveals PAPSS1 contributes to heparan sulfate synthesis
Source: Commun Biol. 2022 Jul 19;5:694. doi: 10.1038/s42003-022-03581-9 (PMC9296583; doi:10.1038/s42003-022-03581-9)
Supplement: Supplementary file 6 — Reporting summary [file 42003_2022_3581_MOESM6_ESM.pdf]

nature research

Corresponding author(s): Yoshitaka Sato

Last updated by author(s): May 6, 2022

Reporting Summary

Nature Research wishes to improve the reproducibility of the work that we publish. This form provides structure for consistency and transparency in reporting. For further information on Nature Research policies, see our Editorial Policies and the Editorial Policy Checklist.

Statistics

For all statistical analyses, confirm that the following items are present in the figure legend, table legend, main text, or Methods section.

n/a	Confirmed	
☐	☒	The exact sample size (*n*) for each experimental group/condition, given as a discrete number and unit of measurement
☐	☒	A statement on whether measurements were taken from distinct samples or whether the same sample was measured repeatedly
☐	☒	The statistical test(s) used AND whether they are one- or two-sided<br>*Only common tests should be described solely by name; describe more complex techniques in the Methods section.*
☒	☐	A description of all covariates tested
☒	☐	A description of any assumptions or corrections, such as tests of normality and adjustment for multiple comparisons
☐	☒	A full description of the statistical parameters including central tendency (e.g. means) or other basic estimates (e.g. regression coefficient) AND variation (e.g. standard deviation) or associated estimates of uncertainty (e.g. confidence intervals)
☒	☐	For null hypothesis testing, the test statistic (e.g. *F*, *t*, *r*) with confidence intervals, effect sizes, degrees of freedom and *P* value noted<br>*Give P values as exact values whenever suitable.*
☒	☐	For Bayesian analysis, information on the choice of priors and Markov chain Monte Carlo settings
☒	☐	For hierarchical and complex designs, identification of the appropriate level for tests and full reporting of outcomes
☒	☐	Estimates of effect sizes (e.g. Cohen's *d*, Pearson's *r*), indicating how they were calculated

Our web collection on statistics for biologists contains articles on many of the points above.

Software and code

Policy information about availability of computer code

Data collection	Illumina HiSeq2500 was controlled by Illumina HiSeq Control software (Illumina). Image Saver 6 (ATTO) was used to acquire blotting images. FACS Canto 2 was controlled by BD FACSDiva software (BD Biosciences). 96-well plate reader was controlled by LS-PLATE manager 2004 (WAKO). 7500 Fast System with 21 CFR Part 11 software (ThermoFisher Scientific) was used to measure viral load. A modular HPLC system (Shimadzu) was operated by LabSolutions LC/GC (Shimadzu).
Data analysis	Enrichment analysis and statistical analyses were performed with Microsoft Excel 2016 MSO 64bit. Indel mutations were analyzed with ApE ver. 2.0.49.0. FACS data was analyzed with FlowJo 7.6.5 (FlowJo Inc). Plaque size measurement was performed with Image J/Fiji v1.53m.

For manuscripts utilizing custom algorithms or software that are central to the research but not yet described in published literature, software must be made available to editors and reviewers. We strongly encourage code deposition in a community repository (e.g. GitHub). See the Nature Research guidelines for submitting code & software for further information.

Data

Policy information about availability of data

All manuscripts must include a data availability statement. This statement should provide the following information, where applicable:

- Accession codes, unique identifiers, or web links for publicly available datasets
- A list of figures that have associated raw data
- A description of any restrictions on data availability

The full list of raw sequencing read counts from the genome-wide CRISPR screen are available in Supplementary Table S6.

Field-specific reporting

Please select the one below that is the best fit for your research. If you are not sure, read the appropriate sections before making your selection.

☒ Life sciences ☐ Behavioural & social sciences ☐ Ecological, evolutionary & environmental sciences

For a reference copy of the document with all sections, see nature.com/documents/nr-reporting-summary-flat.pdf

Life sciences study design

All studies must disclose on these points even when the disclosure is negative.

Sample size	Sample sizes were not predetermined based on statistical methods. Sample sizes were chosen according to previous experience and the standards in the field.
Data exclusions	For plaque area measurement, plaques that contact with other plaque were excluded.
Replication	A genome-wide CRISPR screen was performed in duplicate at the estimated coverage of 150 cells/sgRNA for each mock and HSV-1 infected condition. For viability assay, FACS analysis, progeny virus titer measurement, virus binding assay, cell-to-cell spreading assay and cell proliferation assay, experiments were performed in triplicate for each condition. Quantification of HepS chains was conducted four times for each sample.
Randomization	No human or animal subjects were used in the study. Randomization is not generally used in this field.
Blinding	Blinding is not typically used in this field.

Reporting for specific materials, systems and methods

We require information from authors about some types of materials, experimental systems and methods used in many studies. Here, indicate whether each material, system or method listed is relevant to your study. If you are not sure if a list item applies to your research, read the appropriate section before selecting a response.

Materials & experimental systems

n/a	Involved in the study	
☐	☒	Antibodies
☐	☒	Eukaryotic cell lines
☒	☐	Palaeontology and archaeology
☒	☐	Animals and other organisms
☒	☐	Human research participants
☒	☐	Clinical data
☒	☐	Dual use research of concern

Methods

n/a	Involved in the study	
☒	☐	ChIP-seq
☐	☒	Flow cytometry
☒	☐	MRI-based neuroimaging

Antibodies

Antibodies used	Anti-HepS (10E4 epitope; H1890) mouse monoclonal antibody was purchased from US Biological Life Sciences (Salem, MA, USA). Anti-XYLT2 (G-1; sc-374134), anti-EXT2 (A-2; sc-514092), anti-PAPSS1 (A-2; sc-376244), and normal mouse IgM (sc-3881) antibodies were obtained from Santa Cruz Biotechnology (Dallas, TX, USA). Anti-IRF2BPL (NBP2-56241) antibody was purchased from Novus Biologicals (Littleton, CO, USA). Anti-VANGL2 (clone 2G4; MABN750) antibody was obtained from Merck (Darmstadt, Germany). Anti-GAPDH (D16H11; #5174), horseradish peroxidase-conjugated anti-mouse or anti-rabbit secondary antibodies (#7074 and #7076) were purchased from Cell Signaling Technology (Danvers, MA, USA). Horseradish peroxidase-conjugated anti-rat (SA00001-15) secondary antibody was obtained from Proteintech Group (Rosemont, IL, USA). Alexa Fluor 488-conjugated anti-mouse IgM secondary antibody was obtained from Thermo.
Validation	All antibodies are established, well described and published elsewhere. Informations are accessible on the manufacturers websites under Catalogue numbers.

Eukaryotic cell lines

Policy information about cell lines

Cell line source(s)	HAP1 (# C631; Horizon), HEK293T (CRL-3216; ATCC), RPE-1 (CRL-4000; ATCC), A549 (CCL-185; ATCC), AGS (CRL-1739; ATCC), Vero (CCL-81; ATCC), Huh7 (RCB 1942; RIKEN BRC Cell Bank), HepAD38 cells (a gift from Christoph Seeger, see ref.63)
Authentication	Cell lines from ATCC have been thoroughly tested and authenticated. ATCC uses morphology, karyotyping, and PCR based

Authentication	approaches to confirm the identity of human cell lines and to rule out both intra- and interspecies contamination. https://www.atcc.org/CellAuthenticationMatters.aspx<br>RIKEN BRC CELL BANK has been thoroughly tested and authenticated. RIKEN BRC CELL BANK uses morphology, karyotyping, and PCR based approaches to confirm the identity of human cell lines and to rule out both intra- and inter-species contamination. http://cellbank.brc.riken.jp/cell_bank/CellInfo/?cellNo=RCB4455&lang=Ja
Mycoplasma contamination	Cell lines were not tested for mycoplasma contamination, but no indication of contamination was observed.
Commonly misidentified lines (See ICLAC register)	*Name any commonly misidentified cell lines used in the study and provide a rationale for their use.*

Flow Cytometry

Plots

Confirm that:

☐ The axis labels state the marker and fluorochrome used (e.g. CD4-FITC).

☐ The axis scales are clearly visible. Include numbers along axes only for bottom left plot of group (a 'group' is an analysis of identical markers).

☐ All plots are contour plots with outliers or pseudocolor plots.

☐ A numerical value for number of cells or percentage (with statistics) is provided.

Methodology

Sample preparation	Cells were harvested using the TrypLE Select Enzyme (Sigma-Aldrich), washed with PBS, and subsequently incubated with monoclonal anti-HepS antibody diluted in PBS + 2% FBS. After 1 h of incubation at 4?, the cells were washed with PBS + 2% FBS and incubated with Alexa 488-conjugated secondary antibody. The cells were again washed twice with PBS + 2% FBS.
Instrument	Samples were measured using the FACS Canto2 (BD Biosciences).
Software	FlowJo software (v.7.6.5).
Cell population abundance	Cells were not enriched or sorted.
Gating strategy	We excluded debris to obtain intact cells using FSC/SSC gating. IF staining-positive cells were defined based on negative and positive populations.

☐ Tick this box to confirm that a figure exemplifying the gating strategy is provided in the Supplementary Information.
